# Supplementary figures and images for: Perceived fairness of claimants undergoing a work disability evaluation: Development and validation of the Basel Fairness Questionnaire
Source: PLoS One. 2020 Sep 17;15(9):e0238930. doi: 10.1371/journal.pone.0238930 (PMC7498050; doi:10.1371/journal.pone.0238930)

**BFQ**

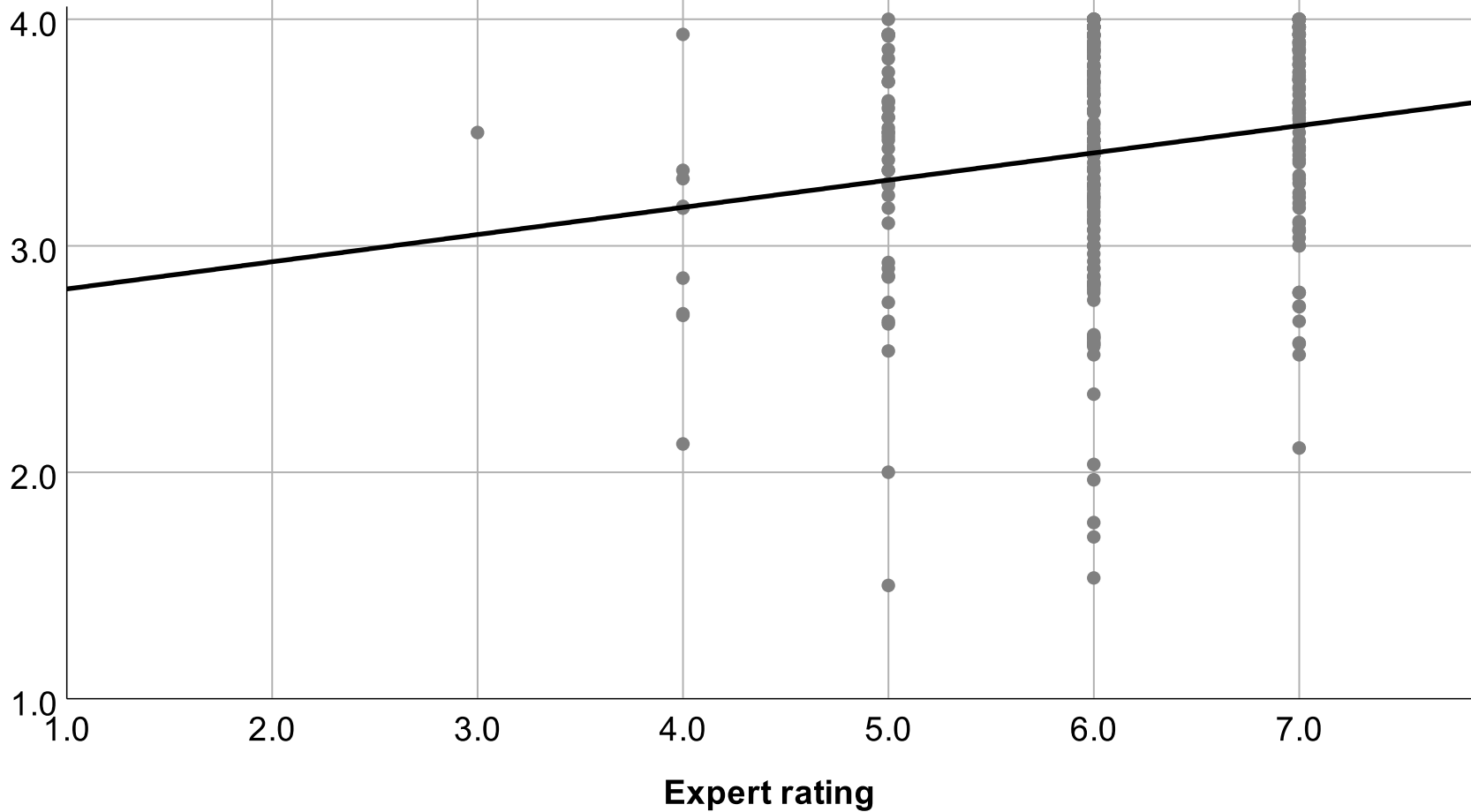

Supplement: S1 Fig — (PDF) [file pone.0238930.s001.pdf]
