# Supplementary material for: Perceived fairness of claimants undergoing a work disability evaluation: Development and validation of the Basel Fairness Questionnaire
Source: PLoS One. 2020 Sep 17;15(9):e0238930. doi: 10.1371/journal.pone.0238930 (PMC7498050; doi:10.1371/journal.pone.0238930)
Supplement: S1 Table — (DOCX) [file pone.0238930.s002.docx]

**Table S1. Rotated factor solution (2 components extracted).**

| **No.** | | **Component** | |
| --- | --- | --- | --- |
|  |  | **1** | **2** |
| 5 | The expert inquired exactly about my complaints. | .880 | -.174 |
| 9 | I felt taken seriously by the expert. | .858 |  |
| 1 | The expert took time for me | .852 | -.100 |
| 10 | The expert took my complaints and restrictions seriously. | .827 |  |
| 6 | I felt understood by the expert. | .800 |  |
| 7 | The expert informed me well. | .726 |  |
| 4 | The expert went into details I told him. | .690 | .140 |
| 11 | The expert responded to me in conversation. | .675 | .196 |
| 13 | The expert valued me. | .674 | .177 |
| 3 | The expert listened to me. | .661 | .115 |
| 15 | The expert treated me respectfully | .616 | .214 |
| 12 | The expert asked me how I feel. | .511 | .232 |
| 16 | I could ask questions. | .404 | .353 |
| 21 | The expert let me finish speaking |  | .878 |
| 22 | I was able to say everything important |  | .806 |
| 25 | The expert looked at me during the evaluation. |  | .747 |
| 28 | The expert involved me in the conservation. |  | .713 |
| 23 | The expert was empathic. | .318 | .557 |
| 24 | The expert paid attention to my complaints and restrictions during the evaluation. | .233 | .509 |
| 30 | The expert could deal with my emotions. | .339 | .496 |
