# Supplementary material for: Perceived fairness of claimants undergoing a work disability evaluation: Development and validation of the Basel Fairness Questionnaire
Source: PLoS One. 2020 Sep 17;15(9):e0238930. doi: 10.1371/journal.pone.0238930 (PMC7498050; doi:10.1371/journal.pone.0238930)
Supplement: Attachment — Submitted filename: Review PONE-D-20-09205.docx [file pone.0238930.s003.docx]

Review PONE-D-20-09205

The authors developed and validated a comprehensive questionnaire aimed at measuring the perceived fairness of disability claimants that underwent a medical work capacity evaluation. The study covers an important and under-researched topic in disability evaluation and approaches recent criticism that have been raised, in particular with regard to the neglect of the claimants view. The questionnaire appears particularly promising as a basis for quality improvement purposes in the evaluation process.

Overall, the paper is interesting and well written and can be accepted pending appropriate revision. There are slight improvement opportunities regarding the English language and a less pedestrian and more direct wording. In addition, the study suffers from an overload of research steps and corresponding detail information presented in the results section. I would suggest thinking about dropping the last two methodological steps (comparison with expert rating, preceding questionnaires) which do not add sufficient robust information on the questionnaire's external validity (or alternatively I suggest reflecting on this in the study limitations section). Moreover, the authors should add a description of the sample characteristics and carefully check that all steps addressed in the results and discussion section are outlined in the methods (i.e. primarily comparison to claimant's preceding evaluations). For suggestions that are more detailed please see below.

ABSTRACT

- You may already introduce the label "Basel Fairness Questionnaire"
- Under methods, you may add that you tested for convergent and divergent validity using the corresponding questionnaire (currently, in the results section of the abstract, these terms appear without being clear what they are referring to)

INTRODUCTION

- Line 54-55: "the legal systems expects that medical experts arrive to similar evaluations for cases that are either similar or identical". – I do not think that any case in disability evaluation is "identical" with another one, so I would suggest that you state "…experts arrive to comparable evaluations for similar cases…"
- Line 57-58: "this lack of quality cannot easily be dissolved…" – "Lack of quality" appears misleading to me, you talk about comparability…
- Line 59: "mental disorders manifest themselves almost exclusively through symptoms" – I would tend to disagree with this statement, mental disorders manifest themselves as limitations in performing work activities and participation restrictions (see Mini ICF-APP by Linden and Baron). I rather see a potential problem with regard to the fact that medical experts in disability evaluation are not sufficiently knowledgeable about limitations in "mental" work activities
- Line 81-82: first sentence of paragraph is incomplete… - suggestion "… might not only be considered as an important quality characteristic of work disability evaluations, but also tend to worsen mental health problems of already vulnerable patients/claimants"

METHODS

- Line 96: "the study started with the collection and creation of items for evaluating patient satisfaction in disability evaluations" – What was the source of the collected items?
- Line 99-100: please introduce the abbreviations for the CPQ and the SWLS, as first mentioned here
- Paragraph study procedure: It is nice that you show an overall view about the methodological steps in your study but I would suggest that – if you do so - you provide complete information. For instance, include the steps about convergent and divergent validity as well as the comparison to preceding questionnaires
- Section BFQ development:

(1) Please provide more details about your literature search (e.g. source databases etc.)
(2) "Colleagues" sounds sloppy – you may replace by "experts" or "key informants"
(3) Subject-matter experts: could you please provide some more information about the experts' professional background and work experience
(4) It appears that the literature search only revealed the Dutch survey and no other candidate instrument for measuring aspects of perceived fairness in disability evaluation – Is this true?
(5) Line 128: Suggest to delete "as published" as no reference needed for the possibility to add verbatim comments
(6) I would suggest to introduce one sentence providing some key information on the CPQ
(7) Line 138: CPQ not CTQ
(8) I have a hard time seeing sense in the step of comparing the claimant ratings of perceived fairness with expert ratings on the same issue. As you correctly point out later on in the discussion section, there is a fundamental social desirability bias and you also measure two fully distinct qualities (an emotional impression by the claimant vs. a cognitive ("objective") impression by the expert) => Therefore using this criteria for "external validity" is – in my view – not methodologically sound
(9) I would clearly point out somewhere in the methods sections that the experts conducting the evaluations were not informed about the fact that their claimants will be invited for a follow-up survey on their perceived fairness of the evaluation

- Section Participants:

Please provide more detailed information about the in- and exclusion criteria. Were German language skills the only exclusion criterion? – In the discussion you refer to additional "exclusion criteria" potentially set by the assessment centers (e.g. vulnerable persons with certain types of personality disorders) => was there a systematic recruitment strategy provided to the assessment centers? In addition, what about individuals with severe neurological disorders (e.g. stroke, traumatic brain injury) who do not have the cognitive capacity for completing the questionnaire? – Were they excluded as well?

RESULTS

- Overall, I think the result part would benefit from reporting less numerical information and focus on the key message
- If available, please provide more information about the sample characteristics including socio-demographics but in particular also information about the claimants' health condition. The latter is crucial for putting your results into context. For instance: how many percentage of the claimants suffered from mental health conditions? / Also presenting some information on the background of the medical experts participating in the study would be helpful.
- In relation to the before-mentioned point you may also provide a non-response analysis to be aware whether responder fundamentally differ from non-responders
- Figure 1: Why does the BFQ mean rating go beyond 4 if the items were to be answered on a scale from 1 to 4?
- Line 214: Language issues… "Missing responses were scare for most items EXCEPT for the four items with the "can't tell" response option…"
- Line 300-306: Here you talk for the first time about the comparison with a preceding evaluation. Please either add the corresponding information to the methods section or delete this study step (which is in my view also charged with a lot of bias, e.g. recall bias) from you’re the results and discussion section of your paper

DISCUSSION

- Overall: please avoid repeating numerical information and details of the results section in the discussion part (e.g. line 380-383)
- Line 317-318: "positive feedback about the perceived fairness in disability evaluations" – please specify "in Switzerland"
- Line 323-325: "we found that participations with good (you mean LOW?) life satisfaction were prone to ratings below the median of the scale, which were virtually absent for participants with good life satisfaction"
- Line 325-26: I think the inference that life situation, life satisfaction and personality have an impact on the satisfaction with disability evaluation is too strong and not directly supported by our findings
- Line 334: "the four assessment centers that participated in the study might reflect the upper quality spectrum". This is a subjective statement without any underlying evidence and I would strongly suggest deleting it – there are various other reasons why other assessment centers may have decided to refrain from participating in the study and not primarily being afraid of their evaluations' quality. I also don't think that you have any evidence in assuming the four participant assessment centers represent the "upper quality level". Moreover, I rather believe that you should acknowledge the focus on this particular four assessment centers of which three are located around Basel as a study limitation with regard to the generalizability of your findings across Switzerland.
- Line 339: Does this mean that the study centers had no clear recruitment instructions and applied their own criteria for excluding particular groups of claimants? (which would be a study limitation)
- Line 347-350: Evaluating the perceived fairness of an evaluation with an interpreter. Would such a questionnaire require additional items or potentially a different item format?
- Line 359-360: In my view this statement is not justified as long as you did not provide a proper analysis of the non-responder sample in your study.
- Line 367-371: This summary graph is – in my view – not necessary and its information may rather be placed at the beginning of the discussion paragraph "mean rating" to outline major points you want to address
- Line 376: You may add expert ratings (and other questionnaires) for testing the external validity of the questionnaire.
- Paragraph line 384 – 396: Would you suggest that the overall BFQ score or the four subscores (or both) should be reported when reporting information on perceived fairness in disability evaluation.
- Line 412-413: Very good and important claim!!!
- Line 430-439: as indicated before comparing expert and claimant ratings is in my view an inappropriate and heavily biased criteria for measuring the questionnaires external validity: (a) emotional vs. cognitive/rational quality of the answers, (b) social desirability and lack of neutrality of experts => a rating of an independent observer would have been a better and less biased criteria (which was however difficult to include with the chosen study design). Just asking the experts more detailed BFQ questions is in my view a first step but not enough.
- Line 440-446: Suggest dropping this paragraph and research step as methodologically unsound and hardly informative.
- Practical implications: Fairness is an important issue in disability evaluation. Your questionnaire measures the perceived fairness with regard to the evaluation process, which is a very important and under researched outcomes. However, there is also another type of "fairness" that is related to the evaluation's output/outcome (including the documentation of statements as well as the work capacity rating itself). Here, aspects like transparency, plausibility, comprehensibility, validity as well as comparability, standardization and objectivity are key criteria. In my view, both a fair process but also a fair reporting and decision process are crucial quality indicators. You slightly touch this issue in your implication section but I suggest to give it a bit more weight (you may also consider a recently conducted study by Schwegler et al. regarding current challenges in disability evaluation at the Swiss Accident Insurance).
- Conclusion: I would suggest that you include a sentence that the BFQ has great potential for quality evaluation and improvement purposes in disability evaluation – a crucial but currently underdeveloped area…
