# Supplementary material for: Perceived fairness of claimants undergoing a work disability evaluation: Development and validation of the Basel Fairness Questionnaire
Source: PLoS One. 2020 Sep 17;15(9):e0238930. doi: 10.1371/journal.pone.0238930 (PMC7498050; doi:10.1371/journal.pone.0238930)
Supplement: Attachment — Submitted filename: BFF_Response letter_30 06 2020.docx [file pone.0238930.s004.docx]

**Response letter**

We greatly appreciated the thoughtful comments of the reviewers and revised the manuscript according to their proposals. All revisions in the manuscript are marked by red font in the track version. Please find the detailed responses to each reviewer comment below.

**Reviewer #1**

The authors developed and validated a comprehensive questionnaire aimed at measuring the perceived fairness of disability claimants that underwent a medical work capacity evaluation. The study covers an important and under-researched topic in disability evaluation and approaches recent criticism that have been raised, in particular with regard to the neglect of the claimants view. The questionnaire appears particularly promising as a basis for quality improvement purposes in the evaluation process.

Overall, the paper is interesting and well written and can be accepted pending appropriate revision. There are slight improvement opportunities regarding the English language and a less pedestrian and more direct wording. In addition, the study suffers from an overload of research steps and corresponding detail information presented in the results section. I would suggest thinking about dropping the last two methodological steps (comparison with expert rating, preceding questionnaires) which do not add sufficient robust information on the questionnaire's external validity (or alternatively I suggest reflecting on this in the study limitations section). Moreover, the authors should add a description of the sample characteristics and carefully check that all steps addressed in the results and discussion section are outlined in the methods (i.e. primarily comparison to claimant's preceding evaluations). For suggestions that are more detailed please see attachment.

ABSTRACT

- You may already introduce the label "Basel Fairness Questionnaire"

As proposed by the reviewer, we now refer to the label in the abstract.

- Under methods, you may add that you tested for convergent and divergent validity using the corresponding questionnaire (currently, in the results section of the abstract, these terms appear without being clear what they are referring to).

We thank the reviewer for noticing this shortcoming. In fact, we did not discuss the convergent and divergent validity in the abstract either. For the sake of clarity and brevity, we removed the sentence referring to the findings on convergent and divergent validity from the abstract.

INTRODUCTION

- Line 54-55: "the legal systems expects that medical experts arrive to similar evaluations for cases that are either similar or identical". – I do not think that any case in disability evaluation is "identical" with another one, so I would suggest that you state "…experts arrive to comparable evaluations for similar cases…"

We acknowledge that each case is unique. However, we refer to the situation when two or more medical experts evaluate the very same patient. We re-worded this section (line 61).

- Line 57-58: "this lack of quality cannot easily be dissolved…" – "Lack of quality" appears misleading to me, you talk about comparability…

The sentence was re-worded, as suggested (line 62).

- Line 59: "mental disorders manifest themselves almost exclusively through symptoms" – I would tend to disagree with this statement, mental disorders manifest themselves as limitations in performing work activities and participation restrictions (see Mini ICF-APP by Linden and Baron). I rather see a potential problem with regard to the fact that medical experts in disability evaluation are not sufficiently knowledgeable about limitations in "mental" work activities.

We thank the reviewer for pointing this out. The sentence was re-worded considering this feedback (line 64).

- Line 81-82: first sentence of paragraph is incomplete… - suggestion "… might not only be considered as an important quality characteristic of work disability evaluations, but also tend to worsen mental health problems of already vulnerable patients/claimants"

The sentence was re-worded, as suggested (line 87).

METHODS

- Line 96: "the study started with the collection and creation of items for evaluating patient satisfaction in disability evaluations" – What was the source of the collected items?

We implemented the paragraph *Study procedure* to provide an overview about the study, not to provide detailed information, as now clarified (line 99). The requested information is provided in the following section.

- Line 99-100: please introduce the abbreviations for the CPQ and the SWLS, as first mentioned here.

Thank you for noticing this flaw. We now introduce the abbreviations (line 103).

- Paragraph study procedure: It is nice that you show an overall view about the methodological steps in your study but I would suggest that – if you do so - you provide complete information. For instance, include the steps about convergent and divergent validity as well as the comparison to preceding questionnaires.

The lack of details is intended. Detailed information is provided in the following paragraphs, as now explicitly stated (line 109).

- Section BFQ development:

(1) Please provide more details about your literature search (e.g. source databases etc.)

We now provide information about the used databases.

(2) "Colleagues" sounds sloppy – you may replace by "experts" or "key informants"

Agreed.

(3) Subject-matter experts: could you please provide some more information about the experts' professional background and work experience.

Thank you for pointing this out. We now shortly describe their work experience (line 123).

(4) It appears that the literature search only revealed the Dutch survey and no other candidate instrument for measuring aspects of perceived fairness in disability evaluation – Is this true?

This is correct and is now explicitly stated (line 113).

(5) Line 128: Suggest to delete "as published" as no reference needed for the possibility to add verbatim comments

The verbatim reports have previously been published, as now clarified (line 135).

(6) I would suggest to introduce one sentence providing some key information on the CPQ

As proposed, we now provide this information (line 145).

(7) Line 138: CPQ not CTQ

We apologize for this typo.

(8) I have a hard time seeing sense in the step of comparing the claimant ratings of perceived fairness with expert ratings on the same issue. As you correctly point out later on in the discussion section, there is a fundamental social desirability bias and you also measure two fully distinct qualities (an emotional impression by the claimant vs. a cognitive ("objective") impression by the expert) => Therefore using this criteria for "external validity" is – in my view – not methodologically sound.

For the reasons outlined by the reviewer, we do not to label the association between claimant and expert ratings as criterion for external validity any further.

(9) I would clearly point out somewhere in the methods sections that the experts conducting the evaluations were not informed about the fact that their claimants will be invited for a follow-up survey on their perceived fairness of the evaluation.

This is not fully correct. Medical experts were informed about the study intent and were aware that the claimants rated their performance, as now clarified. This awareness might have affected the expert’s behavior during the evaluation, as now discussed (*Hawthorne* or observer effect, line 397).

- Section Participants:

Please provide more detailed information about the in- and exclusion criteria. Were German language skills the only exclusion criterion? – In the discussion you refer to additional "exclusion criteria" potentially set by the assessment centers (e.g. vulnerable persons with certain types of personality disorders) => was there a systematic recruitment strategy provided to the assessment centers? In addition, what about individuals with severe neurological disorders (e.g. stroke, traumatic brain injury) who do not have the cognitive capacity for completing the questionnaire? – Were they excluded as well?

Yes, the lack of sufficient German language skills and age below 18 years were the only exclusion criteria. The assessment centers were supposed to send the BFQ to all eligible claimants. Due to the anonymization of the questionnaires, we have no medical diagnostic information and other personal data of the participants at hand, except the demographic data provided by the claimants. Medical diagnostic information of all claimants *invited* to participate would have allowed substantiating our speculation that certain claimants (such as vulnerable persons with certain types of personality disorders) did not receive the questionnaire. Moreover, such data would have allowed a more detailed analysis of invited claimants who did not return the questionnaire. Data safety regulations and the ethics committee approval did not allow the provision of medical diagnostic data by the assessment centers to the research team, as now discussed (line 360).

Individuals with severe neurological disorders were not per se excluded from participation, but such individuals are unlikely to undergo multidisciplinary medical evaluations (because monodisciplinary medical evaluations are in general sufficient for claimants with severe neurological disorders).

RESULTS

- Overall, I think the result part would benefit from reporting less numerical information and focus on the key message.

Following this advice, we removed some less relevant information from Results (rating of previous evaluation).

- If available, please provide more information about the sample characteristics including socio-demographics but in particular also information about the claimants' health condition. The latter is crucial for putting your results into context. For instance: how many percentage of the claimants suffered from mental health conditions?

Unfortunately, we cannot provide these details. The questionnaire asked for some basic demographic data of participants (age, sex, marital status). Beyond that, the research team had no access to medical and other records of the participants, as now explicitly mentioned in Methods (line 175) and discussed as study limitation (line 362).

- Also presenting some information on the background of the medical experts participating in the study would be helpful.

The expert questionnaire did not ask for any information on the expert in order to assure the anonymization of the expert data. The claimants were asked to name the discipline, in which the evaluation took place, as now mentioned in Methods (line 175). Unfortunately, a considerable number of participants did not name the discipline or named more than one discipline, meaning their responses were ambiguous. We only analysed the ratings across all disciplines for methodological reasons (primarily because of the limited sample size).

- In relation to the before-mentioned point you may also provide a non-response analysis to be aware whether responder fundamentally differ from non-responders.

The research team had no access to medical and other records of claimants, as outlined above. Unfortunately, this also means that we do not have any data on claimants who did not return the questionnaire.

- Figure 1: Why does the BFQ mean rating go beyond 4 if the items were to be answered on a scale from 1 to 4?

Our apologies for this misleading impression. The mean ratings did not go beyond 4, as also evident from Figures 2 to 4. Figure 1 was revised to avoid any misreading of the data.

- Line 214: Language issues… "Missing responses were scare for most items EXCEPT for the four items with the "can't tell" response option…"

The sentence was re-worded, as requested.

- Line 300-306: Here you talk for the first time about the comparison with a preceding evaluation. Please either add the corresponding information to the methods section or delete this study step (which is in my view also charged with a lot of bias, e.g. recall bias) from you’re the results and discussion section of your paper).

Following the reviewer’s request, we removed the description and discussion of these indeed not very relevant results.

DISCUSSION

- Overall: please avoid repeating numerical information and details of the results section in the discussion part (e.g. line 380-383)

Numerical information already provided in Results was removed from Discussion.

- Line 317-318: "positive feedback about the perceived fairness in disability evaluations" – please specify "in Switzerland"

We specified the information as requested.

- Line 323-325: "we found that participations with good (you mean LOW?) life satisfaction were prone to ratings below the median of the scale, which were virtually absent for participants with good life satisfaction"

We thank the reviewer for pointing out this error (line 345).

- Line 325-26: I think the inference that life situation, life satisfaction and personality have an impact on the satisfaction with disability evaluation is too strong and not directly supported by our findings.

We agree and toned down the wording of this sentence (line 346).

- Line 334: "the four assessment centers that participated in the study might reflect the upper quality spectrum". This is a subjective statement without any underlying evidence and I would strongly suggest deleting it – there are various other reasons why other assessment centers may have decided to refrain from participating in the study and not primarily being afraid of their evaluations' quality. I also don't think that you have any evidence in assuming the four participant assessment centers represent the "upper quality level". Moreover, I rather believe that you should acknowledge the focus on this particular four assessment centers of which three are located around Basel as a study limitation with regard to the generalizability of your findings across Switzerland.

The statement is indeed not supported by any empirical data. In consequence, we re-worded the section, considering the reviewer’s feedback (line 356).

- Line 339: Does this mean that the study centers had no clear recruitment instructions and applied their own criteria for excluding particular groups of claimants? (which would be a study limitation)

No, this was somewhat misleading. The critical point (and study limitation) is that the ethic committee approval covered only the collection of the questionnaire data, which means that no demographic or medical data of participants and non-participants were available, except the demographic data provided by the participants on the questionnaire. This considerably limits the characterization of the study sample, but also prohibits the characterization of non-participants (claimants who were either not invited to participate or who were invited, but did not return the questionnaire). Given this, we cannot rule out that the study sample varied from non-responders and was, thus, not representative for claimants with good German command in general. We re-worded the section (line 360) and already mention in Methods that we had no access to clinical records of participants.

- Line 347-350: Evaluating the perceived fairness of an evaluation with an interpreter. Would such a questionnaire require additional items or potentially a different item format?

Currently, we assume that such a questionnaire would not require additional items or a different item format. If future research shows that work disability evaluations with interpreter are perceived as less fair than work disability evaluations without interpreter, a different research approach will be needed to attribute these deficiencies to a cause.

- Line 359-360: In my view this statement is not justified as long as you did not provide a proper analysis of the non-responder sample in your study.

We agree with the reviewer that this statement is not sufficiently supported by the data. The statement (“We therefore consider selection bias by non-responders as the least important source of bias. “) was consequently removed.

- Line 367-371: This summary graph is – in my view – not necessary and its information may rather be placed at the beginning of the discussion paragraph "mean rating" to outline major points you want to address.

The paragraph was intended to provide a summary of why the positive ratings should interpreted with some caution. We re-worded the paragraph to make this intention clearer (line 401).

- Line 376: You may add expert ratings (and other questionnaires) for testing the external validity of the questionnaire.

Following the reviewer’s argumentation further above, we do not any longer refer to expert ratings as criterion of external validity. In consequence, the discussion of the expert ratings is now a short section on its own. For better overview, we re-worded the initial paragraph of the discussion, now listing all sections of the discussion (line 331).

- Paragraph line 384 – 396: Would you suggest that the overall BFQ score or the four subscores (or both) should be reported when reporting information on perceived fairness in disability evaluation.

Yes, this is indeed what we propose, as now explicitly stated (line 429).

- Line 412-413: Very good and important claim!!!

We thank the reviewer for this kind feedback. We think (and propagate) that quality control and quality improvement are in the best interest of all stakeholders.

- Line 430-439: as indicated before comparing expert and claimant ratings is in my view an inappropriate and heavily biased criteria for measuring the questionnaires external validity: (a) emotional vs. cognitive/rational quality of the answers, (b) social desirability and lack of neutrality of experts => a rating of an independent observer would have been a better and less biased criteria (which was however difficult to include with the chosen study design). Just asking the experts more detailed BFQ questions is in my view a first step but not enough.

We principally agree with the reviewer’s argumentation. However, using independent observers for rating the expert’s interviewing performance is likely only suited for expert training and it would be restricted to a limited number of evaluations (for legal and cost efficiency reasons). Aside of the advantage that expert self-rating could principally be obtained in any work disability evaluation with little additional workload for the expert, such ratings turn the expert’s attention to the relevant dimensions of perceived fairness and allow the expert to reflect and to self-monitor his interview performance.

- Line 440-446: Suggest dropping this paragraph and research step as methodologically unsound and hardly informative.

The paragraph was dropped, as suggested.

- Practical implications: Fairness is an important issue in disability evaluation. Your questionnaire measures the perceived fairness with regard to the evaluation process, which is a very important and under researched outcomes. However, there is also another type of "fairness" that is related to the evaluation's output/outcome (including the documentation of statements as well as the work capacity rating itself). Here, aspects like transparency, plausibility, comprehensibility, validity as well as comparability, standardization and objectivity are key criteria. In my view, both a fair process but also a fair reporting and decision process are crucial quality indicators. You slightly touch this issue in your implication section but I suggest to give it a bit more weight (you may also consider a recently conducted study by Schwegler et al. regarding current challenges in disability evaluation at the Swiss Accident Insurance).

We greatly appreciate this comment and could not agree more. The paragraph was slightly extended, considering the reviewer comment (line 490).

- Conclusion: I would suggest that you include a sentence that the BFQ has great potential for quality evaluation and improvement purposes in disability evaluation – a crucial but currently underdeveloped area…

As proposed, we added such a sentence to Conclusions (line 499).

**Reviewer #2:**

The paper presents novel research that aims to measure the claimant experience, specifically perceived fairness, of disability evaluations. The research has been well justified, and the design and methodology used seemed appropriate. The results well explained and outlined in detail.

We thank the reviewer for this kind feedback.

I had only a few queries about the development of the questionnaire and recruitment of participants and medical expert role. There is little detail explanation about the "subject-matter experts", and how they influenced the dimensions of the questionnaire. On the questionnaire, the principles of informational and interactive justice can easily be recognised in the questions, yet it is difficult to see how procedural justice is measured.

It may be helpful for international readers to give a brief explanation of the Swiss disability evaluation process and note whether it is mandatory for claimants to attend a disability assessment.

We thank the reviewer for pointing this out. Following this request, we now provide such short explanation and state that the attendance of such medical evaluations is obligatory for claimants (line 48 and 164).

Might mandatory attendance influence the claimant to rate the evaluation positively if they feared a negative outcome?

The study information stressed that the questionnaire data were fully anonymized and that the responses in the questionnaire would in consequence have no impact on the outcome of the disability evaluation. Thus, the study design emphasized that claimants were fully free in their responses. However, the dyadic interaction between medical expert and claimant is asymmetric, with one party evaluating and the other party being evaluated. Claimants as the evaluated party might see themselves in a subordinate position and, therefore, might find it difficult or even risky to provide negative feedback to medical experts, even though anonymity is granted. This is now outlined in the discussion (line 386).

It is unclear how the assessment centres or medical experts were chosen or enrolled in the study. Also how soon after examining a claimant did the medical experts complete their ratings? Were the medical experts aware the claimants were rating their performance?

The study aimed to collect 300 questionnaires from claimants within a reasonable amount of time. Inclusion of additional assessment centres would presumably have shortened the time to collect the data, but would also have increased the organizational workload. Therefore, we did not seek to increase the number of assessment centres after four assessment centres signaled their readiness to participate. Within the assessment centres, we had no influence on selecting the medical experts/disciplines for the study. The assessment centres sought to distribute the (relatively small additional) workload resulting from study participation equally across the contracted medical experts. The distribution of disciplines, in which the evaluation took place, is now provided in Results (line 220). We only analysed the ratings across all disciplines for methodological reasons.

Medical experts had to return their rating immediately after the evaluation. They were informed about the study intent and were aware that the claimants rated their performance, as now clarified. This awareness might have affected the expert’s behavior during the evaluation, as now discussed (*Hawthorne* or observer effect, line 394).

Why were 12 claimants not rated?

The return rate of expert ratings was fortunately very high. We do not know why expert ratings were not returned in rare cases. It might have had trivial reasons: the questionnaire might for example have been stashed with other documents before it could be returned.

Overall the limitations of the research, and potential mechanisms for redress were well considered and thoroughly discussed. The recommendations for further study and evaluation of the questionnaire are also logical and commendable. With the further testing and refinement proposed by the authors, it seems this questionnaire could be used as a valuable feedback mechanism to inform and improve medical expert performance and thus the quality of assessment. This should then also benefit claimant experience. Creation of a tool to measure the fairness of disability evaluations is an important contribution to the field and potentially has applicability across jurisdictions.

We greatly appreciate this favorable assessment.
