# Supplementary material for: Perceived fairness of claimants undergoing a work disability evaluation: Development and validation of the Basel Fairness Questionnaire
Source: PLoS One. 2020 Sep 17;15(9):e0238930. doi: 10.1371/journal.pone.0238930 (PMC7498050; doi:10.1371/journal.pone.0238930)
Supplement: Attachment — Submitted filename: BFF_Response letter_25 08 2020.docx [file pone.0238930.s006.docx]

We thank the editor and reviewers for their helpful comments. We greatly appreciated the comments made by the editor. All revisions in the text are marked by red font.

**Editor**

Lines 88 and 491 Please do not use the term "accidents". You could reword this sentence to just read "after sustaining musculoskeletal injuries".

We re-worded the sentence and do not use the term accident any more.

Line 115 reword to "as a starting"

Done.

Line 234 This appears to be an error?

This was no error, but unnecessarily complex. We simplified the sentence.

Line 238 Please make sure that all tables are formatted consistently using the PLoS ONE instructions to authors.

We re-checked the formatting and removed unnecessary formatting. We re-formatted the labels and titles.

Line 312 Instead of saying "mean" or "median" of the scale, which should refer to the distribution of responses, provide the semantic label and value for the "middle" possible score on the questionnaire?

We now explain that such SWLS ratings indicate that the participants tended to be rather dissatisfied with their lives.

Line 324 Is this the mean value?

Yes. We now clarify that the reported values represent mean values.

Line 338 As above, provide the semantic label?

We now explain that such BFQ ratings indicate that only 4 % of the participants tended to perceive the disability evaluation as unfair.

Line 348 I'm not sure this is the correct wording?

The wording was changed.

Line 354 Please fix this wording.

Done.

Line 399 delete "the".

Done.
